# Types and spatial distribution of cancer cases registered in a national hospital-based registry in the Lao People’s Democratic Republic from 2010 to 2024

**DOI:** 10.1186/s12889-026-27554-3

**Published:** 2026-04-28

**Authors:** Champadeng Vongdala, Oraya Sahat, Thitsamay Luangxay, Apisit Chaidee, Kitti Intuyod, Supot Kamsa-ard, Somchai Pinlaor

**Affiliations:** 1 National Cancer Center, Vientiane Capital, Lao People’s Democratic Republic, Vientiane, Laos; 2 Tha Uthen District Public Health Office, Nakhon Phanom, Thailand; 3 Department of Parasitology, Faculty of Medicine, Khon Kaen University, Khon Kaen, 40002 Thailand; 4 Department of Pathology, Faculty of Medicine, Khon Kaen University, Khon Kaen, 40002 Thailand; 5 Department of Epidemiology and Biostatistics, Faculty of Public Health, Khon Kaen University, Khon Kaen Province, 40002 Thailand; 6 Cholangiocarcinoma Research Institute, Faculty of Medicine, Khon Kaen University, Khon Kaen, 40002 Thailand

**Keywords:** Spatial distribution, Cancer, National Cancer Centre, Lao People's Democratic Republic

## Abstract

**Background:**

The Lao People’s Democratic Republic (Lao PDR) is experiencing an epidemiological transition with a rising burden of non-communicable diseases, including cancer. However, the absence of a national population-based cancer registry (PBCR) limits the understanding of the true cancer burden and its geographical distribution, hindering effective public health planning. We present the first spatial analysis of hospital-based cancer data in Lao PDR to address this critical knowledge gap.

**Methods:**

We conducted a descriptive retrospective analysis of cancer cases recorded at the National Cancer Center from January 1, 2010, to December 31, 2024. Data on patient demographics, basis of diagnosis, and primary cancer site (ICD-10) were extracted and analyzed. Descriptive statistics were used to summarize patient characteristics, and spatial analysis was performed to map the geographic distribution of cancer cases at the district level across different time periods.

**Results:**

A total of 1,738 cancer cases were analyzed, with a female/male ratio of 1.76 (1,108/630). The most frequent malignancy in males was liver and bile-duct cancer (15.1%), while cervical cancer was predominant in females, accounting for 41.1% of cases. Reported cases were heavily concentrated in Vientiane province and surrounding central provinces throughout the study period, suggesting under-reporting elsewhere. However, case detection in northern and southern provinces showed marked improvement in the most recent period (2020–2024), suggesting a strengthening of national surveillance capabilities. Cancer types varied in frequency across Laos, with cervical cancer cases being widely distributed.

**Conclusion:**

This study reveals that Lao PDR faces a significant burden of cervical and liver cancers, with reported cases heavily concentrated in Vientiane. Despite recent surveillance improvements, the limitations mentioned above underscore the urgent need for a national PBCR for effective cancer control.

## Background

The Lao People’s Democratic Republic (Lao PDR) has undergone a profound epidemiological transition, marked by a decline in the burden of communicable diseases and the concomitant rise of non-communicable diseases (NCDs), with cancer emerging as a formidable public health threat [1–3]. The cancer burden has escalated significantly, ranking as the fifth leading cause of morbidity and mortality, measured as disability-adjusted life years (DALYs) in 2019 and accounting for an estimated 2,200 DALYs lost per 100,000 population [2, 4–6]. Projections from the Global Cancer Observatory (GLOBOCAN) 2020 underscore the scale of the challenge, estimating 9,133 new cancer cases and 6,208 cancer-related deaths annually from the total population of 7.2 million. Among cancer cases, liver cancer (19.2%) and lung cancer (14.4%) represent the leading causes of cancer-related mortality [6, 7]. This high burden reflects a multifaceted etiology driven by a combination of infectious agents and lifestyle-related risk factors [8–10]. For example, key risk factors for liver cancer include infection-related causes, such as hepatitis B and C viruses and liver fluke (*Opisthorchis viverrini*) infection, as well as lifestyle-related issues, including high alcohol consumption [11]. In contrast, human papillomavirus (HPV) is the primary cause of cervical cancer [12].

The cornerstone of any effective national cancer control strategy is a robust surveillance system, for which a population-based cancer registry (PBCR) is the international gold standard [13]. PBCRs can provide comprehensive, high-quality data on cancer occurrence, which is essential for accurately estimating disease burden, monitoring trends, and planning evidence-based interventions [13, 14]. A critical infrastructure and research gap exists in the Lao PDR: the country has no functioning national PBCR. This deficit forces premier global health agencies, including the International Agency for Research on Cancer (IARC), to rely on statistical models extrapolated from neighboring countries to estimate the nation’s cancer burden. This methodology, while necessary, risks obscuring the unique epidemiological and socio-demographic characteristics of the Lao population [15, 16].

In the absence of a national PBCR, Lao PDR depends on one institution-based data source, a single hospital-based cancer registry (HBCR) [15]. While representing crucial progress, these systems face significant operational and financial challenges that limit their scope and data quality. For instance, the pathology registry captures only some of the estimated national cancer-case incidence, while the HBCR is confined to a single tertiary center and struggles with a high proportion of cases with an unknown primary site (> 20%) [13, 14]. These limitations reflect a fundamental structural issue: institution-based systems designed for clinical documentation lack the dedicated infrastructure, standardized protocols, and trained registry personnel required for true cancer surveillance. Without systematic case ascertainment and quality control mechanisms, such facilities can only capture patients who reach their doors—often at advanced stages when primary sites may be difficult to determine. This results in an incomplete and potentially biased picture of the true cancer burden and its distribution.

Despite these structural constraints, these institution-based data sources remain the only available records of direct, patient-level cancer information in Lao PDR. To date, there has been no attempt to understand the geographic dimension of the disease. Spatial analysis offers a powerful methodology to transform this fragmented, service-based data into actionable public-health intelligence. This spatial approach can partially compensate for the absence of population-based surveillance by revealing which communities are—or critically, are not—accessing cancer services, providing essential baseline intelligence for eventually establishing a functional national registry system. For a country with diverse geography and infrastructure challenges like Lao PDR, such insights are indispensable to support objective resource allocation, targeted public-health messaging, and strategic health-service planning.

Therefore, this study aims to conduct the first spatial distribution analysis of hospital-based cancer record data in the Lao PDR. The primary objective is to describe and analyze the geographic distribution of registered cancer cases, providing foundational evidence to inform targeted interventions, guide resource allocation, and shape the future of the national cancer control strategy.

## Methods

### Study area and design

**Fig. 1:**
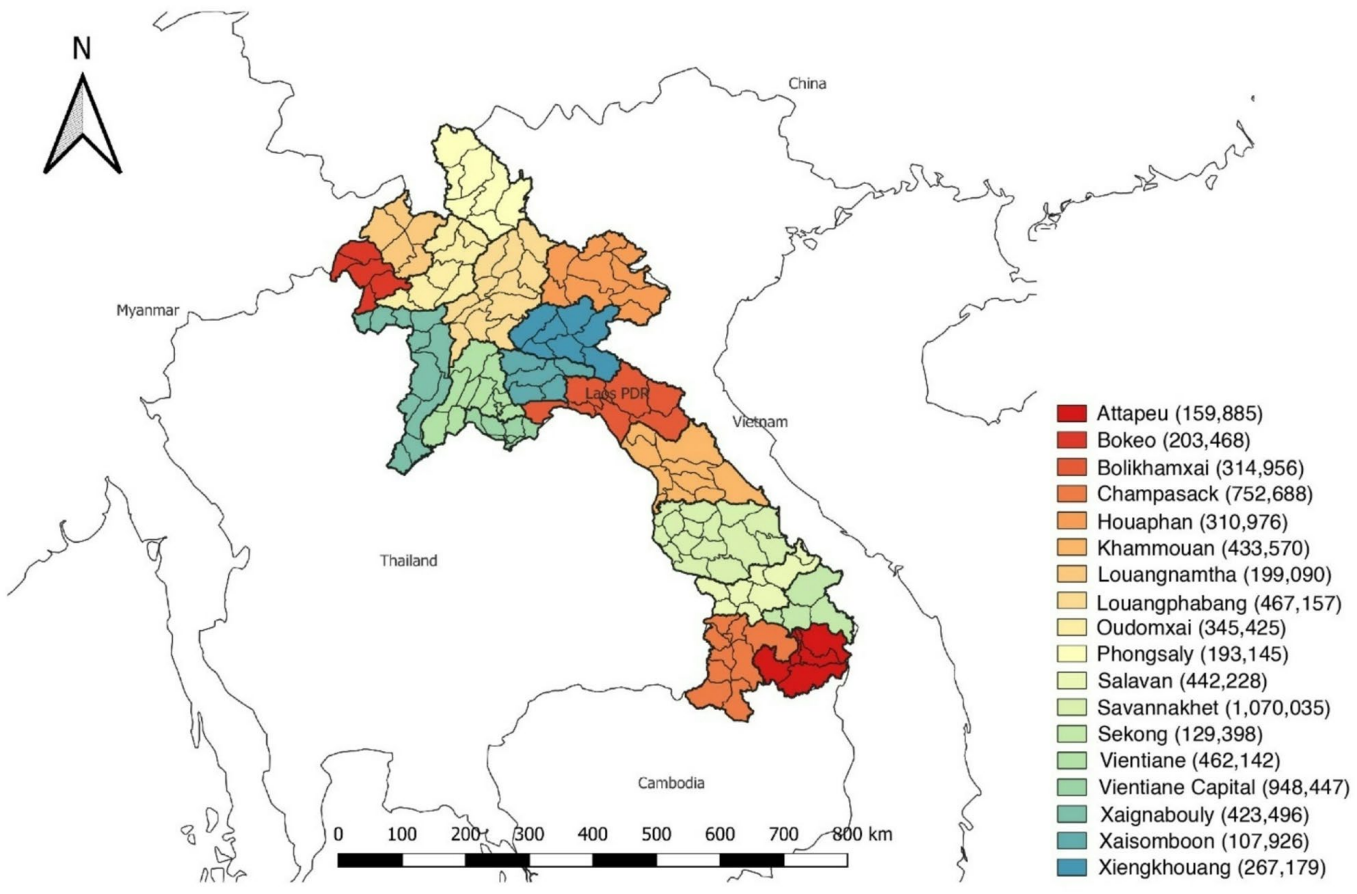
The map of provinces in Laos. Population by province is indicated in parentheses (2020 data, https://www.citypopulation.de/en/laos/admin/)

Laos is administratively divided into 17 provinces plus the Vientiane Capital city containing 148 districts overall, as shown in Fig. 1. As of 2020, the total population stands at 7,231,210, with a nearly equal distribution between males (3,622,996) and females (3,608,214) (https://www.citypopulation.de/en/laos/admin/). This descriptive retrospective study was based on cancer records over 15 years (2010–2024) from the cancer patients who registered in the Cancer Center Unit based at Mittaphab Hospital, Vientiane.

### Data collection

Cancer cases recorded in the oncology clinic registry between January 1, 2010, and December 31, 2024, were assessed for coverage quality. Relevant information was extracted from standardized hard-copy forms and categorized into three sections: (a) Demographics (sex, date of birth, age at diagnosis, date of diagnosis, address [at district level]); (b) Basis of Diagnosis (histology of primary tumor, histology of metastasis, cytology, specific biochemical/immunological tests, surgery/autopsy without histology, endoscopy/radiology, history and physical examination, or death certificate only: DCO); and (c) Diagnosis Code (coded according to the International Classification of Diseases for Oncology, 3rd Edition: ICD-O-3).

### Data management

Patient age was categorized into 16 groups: those aged 0–14 years were combined into a single category, those aged 15–84 years were divided into 5-year intervals, and those aged 85 years and above formed the final category. A total of 1,738 patients diagnosed with cancer were evaluated for accuracy and potential duplications. Patient district codes were used to map cases to individual districts.

Each registered cancer patient’s place of residence was recorded at the time of diagnosis as part of the standard registration process. Patient address codes corresponding to district-level administrative units were used to geocode and assign each case to its respective district. The number of cancer cases per district was then aggregated and merged with the district-level boundary data using the district identifier as the linking key. This patient-to-map linkage allowed the spatial distribution of cancer cases to be visualized across all districts of Laos. Cases were mapped across three time periods (2010–2014, 2015–2019, and 2020–2024) to illustrate temporal trends in case reporting and geographic coverage. Districts with no recorded cases were displayed in gray, while those with recorded cases were color-coded according to the number of cases, ranging from green (few cases) to red (higher number of cases), using a continuous color scale. Universal Transverse Mercator (UTM) coordinates were used as the projection system, with northings and eastings expressed in kilometers (km).

### Data analysis

The geographic boundary data for Laos, including province- and district-level administrative boundaries, were obtained from publicly available shapefiles provided by the Database of Global Administrative Areas (GADM; https://gadm.org). The map covered all 17 provinces and Vientiane Capital city, encompassing 148 districts in total. Spatial data were imported into R (version 4.4.3) using the maptools package for reading and importing spatial vector data, and the ggplot2 package was used to render and visualize the geographic distribution of cancer cases.

## Results

### Patient characteristics

Over the 15 years (2010–2024), 1,738 cancer cases were recorded. The female/male ratio was 1.76 (1,108/630). The mean age of patients at diagnosis was 53.81 for males and 48.16 for females. Cancer cases peaked in older age groups for males than for females. In males, the 55–59 and 60–64 age groups each accounted for 15.40% of cases (*n* = 97 per group). Conversely, female cases were most prevalent in the 45–49 (15.43%, *n* = 171) and 50–54 (14.26%, *n* = 158) age cohorts (Table 1).

**Table 1:** Characteristics of study participants at the time of diagnosis in Laos from 2010 to 2024

Characteristics	Males		Females	
	Number (*n* = 630)	%	Number (*n* = 1,108)	%
Age at diagnosis (year)				
0–14	2	0.32	2	0.18
15–19	10	1.59	10	0.90
20–24	8	1.27	22	1.99
25–29	20	3.17	47	4.24
30–34	30	4.76	91	8.21
35–39	41	6.51	121	10.92
40–44	43	6.83	144	13.00
45–49	71	11.27	171	15.43
50–54	64	10.16	158	14.26
55–59	97	15.40	117	10.56
60–64	97	15.40	99	8.94
65–69	73	11.59	66	5.96
70–74	36	5.71	32	2.89
75–79	28	4.44	18	1.62
80–84	5	0.79	7	0.63
85+	5	0.79	3	0.27
Mean (Standard deviation)	53.81 yrs. (14.48 yrs.)		48.16 yrs. (13.22 yrs.)	
Median (Minimum: Maximum)	56 yrs. (3:95 yrs.)		48 yrs. (5 yrs. : 96 yrs.)	
Basis of diagnosis				
History and physical examination	3	0.32	10	0.90
Endoscopy and radiology	121	19.24	81	7.31
Surgery and autopsy (no histology)	5	0.79	4	0.36
Specific biochemical/ immunological tests	5	0.79	8	0.72
Hematologic cytology	15	2.38	19	1.71
Histology of metastasis	21	3.34	31	2.80
Histology of primary	459	72.97	952	85.92
Autopsy with histology	-	0.00	3	0.27
Unknown	1	0.16	-	-

*n* Number of cancer cases

Table 1 shows diagnostic methods used in medical cases arranged by gender. “Histology of primary” was the most common method, 459 cases (72.97%) in males and 952 cases (85.92%) in females. “Endoscopy and radiology” were the second most common, especially for males: 121 cases (19.24%) in males versus only 81 cases (7.31%) in females. All other diagnostic methods (History and physical examination, Surgery and autopsy (no histology), Specific biochemical/immunological tests, Hematologic cytology, Histology of metastasis, Autopsy with histology, Unknown) were much less frequent, each representing under 4% of cases in both sexes. Histological diagnoses were much more frequent for females.

### Cancer distribution by primary site

Table 2 presents male cancer cases. Liver and bile-duct cancer was the largest category, with 95 cases (15.10%), followed by colon & rectum cancer with 72 cases (11.45%), trachea, bronchus and lung (51 cases; 8.11%), then nasopharynx cancer (50 cases; 7.95%), stomach cancer (43 cases; 6.84%), and oral-cavity cancer (40 cases; 6.36%).

**Table 2:** Number of male cancer cases reported in the period 2010 to 2024, arranged by primary site and age group

Site	ICD-10	Age group																Total	%
		0–14	15–19	20–24	25–29	30–34	35–39	40–44	45–49	50–54	55–59	60–64	65–69	70–74	75–79	80–84	85+		
Oral cavity	C00-C08	-	-	-	3	2	3	3	8	3	5	8	3	-	1	1	-	40	6.36
Oropharynx	C09-C10, C14	-	-	-	-	2	1	2	3	1	-	2	2	2	-	-	-	15	2.38
Nasopharynx	C11	-	-	1	3	5	4	6	2	4	4	7	11	2	1	-	-	50	7.95
Hypopharynx	C12-C13	-	-	-	-	-	-	-	-	-	1	-	-	-	-	-	-	1	0.16
Esophagus	C15	-	-	-	-	-	-	-	-	-	3	1	1	1	1	-	-	7	1.11
Stomach	C16	-	-	-	-	1	3	3	6	5	5	5	8	4	2	1	-	43	6.84
Small intestine	C17	-	1	-	-	-	-	-	1	-	-	-	-	-	-	-	-	2	0.32
Colon & Rectum	C18-C21	-	-	1	3	4	5	3	11	4	17	12	7	4	1	-	-	72	11.45
Liver and bile duct	C22, C24	-	-	-	-	2	-	4	11	19	17	12	14	9	6	-	1	95	15.10
Gallbladder	C23	-	-	-	-	-	-	-	-	-	-	-	-	-	1	-	-	1	0.16
Pancreas	C25	-	-	-	1	-	-	-	-	1	-	-	-	-	-	-	-	2	0.32
Nose, sinuses etc.	C30-C31	-	1	-	-	-	1	1	-	1	2	1	2	-	-	-	1	10	1.59
Trachea, bronchus and lung	C33-C34	-	-	-	-	-	3	3	6	4	6	12	7	7	3	-	-	51	8.11
Other thoracic organs	C37-C38	-	-	-	-	2	-	-	-	-	1	-	-	-	-	-	-	3	0.48
Bone	C40-C41	1	5	-	-	2	-	1	1	-	-	1	-	-	-	1	-	12	1.91
Other skin	C44	-	1	2	1	1	4	2	5	4	5	5	4	1	3	-	1	39	6.20
Connective and soft tissue	C47, C49	-	-	-	2	2	1	3	4	2	4	-	1	-	2	-	1	22	3.50
Breast	C50	-	-	-	-	-	-	-	1	2	1	-	-	-	-	-	-	4	0.64
Penis	C60	-	-	-	-	-	-	2	1	2	1	3	3	-	-	-	-	12	1.91
Prostate	C61	-	-	-	-	-	-	-	-	-	4	5	1	2	5	2	-	19	3.02
Testis	C62	-	-	3	1	1	1	-	-	-	2	2	-	-	-	-	-	10	1.59
Other male genital organs	C63	-	-	-	-	-	-	-	-	-	-	1	-	-	-	-	-	1	0.16
Kidney	C64	-	-	-	1	1	1	1	2	1	3	-	2	-	-	-	-	12	1.91
Renal pelvis	C65	-	-	-	-	-	-	-	-	1	-	-	1	-	-	-	-	2	0.32
Bladder	C67	-	-	-	-	1	-	-	1	1	4	3	-	2	-	-	-	12	1.91
Other urinary organs	C68	-	-	-	-	-	-	-	-	-	-	1	-	-	-	-	-	1	0.16
Eye	C69	1	-	-	-	-	-	-	1	-	-	-	-	-	-	-	-	2	0.32
Brain, nervous system	C70-C72	-	-	-	-	-	4	2	-	1	-	3	-	-	-	-	-	10	1.59
Thyroid	C73	-	1	-	-	1	1	-	1	-	3	-	1	-	-	-	-	8	1.27
Other and unspecified	O&U	-	1	1	4	4	9	7	6	8	9	13	5	2	2	-	1	71	11.29
	Total	2	10	8	19	31	41	43	71	64	97	97	73	36	28	5	5	630	100

Cervical cancer was by far the most common form among females (455 cases; 41.06%). There were 177 cases of breast cancer (15.97%), followed by liver and bile-duct cancer (57 cases; 5.14%). Other cancers, such as those of the nasopharynx (24 cases, 2.17%) and ovaries (24 cases, 2.17%), were less frequent (Table 3).

**Table 3:** Number of female cancer cases reported in the period 2010 to 2024 arranged by primary site and age group

Site	ICD-10	Age group																Total	%
		0–14	15–19	20–24	25–29	30–34	35–39	40–44	45–49	50–54	55–59	60–64	65–69	70–74	75–79	80–84	85+		
Oral cavity	C00-C08	-	-	2	-	-	2	2	5	10	5	3	4	2	-	2	-	37	3.34
Oropharynx	C09-C10, C14	-	-	-	-	-	1	2	2	-	-	1	-	-	-	-	-	6	0.54
Nasopharynx	C11	-	-	-	4	1	-	5	5	6	1	-	-	1	1	-	-	24	2.17
Esophagus	C15	-	-	-	-	-	-	-	-	-	1	1	-	-	-	-	-	2	0.18
Stomach	C16	-	-	-	-	3	3	-	1	1	3	1	2	1	-	-	-	15	1.35
Colon & Rectum	C18-C21	-	1	1	2	2	4	3	6	10	7	9	3	3	1	1	-	53	4.78
Liver and bile duct	C22, C24	-	-	1	2	-	3	1	9	6	9	15	4	3	4	-	-	57	5.14
Gallbladder	C23	-	-	-	-	-	-	2	-	-	2	-	-	-	-	-	-	4	0.36
Pancreas	C25	-	-	-	-	-	-	-	-	1	-	-	-	-	-	1	-	2	0.18
Nose, sinuses etc.	C30-C31	-	-	-	-	-	2	2	2	-	-	-	-	-	-	-	-	6	0.54
Larynx	C32	-	-	-	-	-	-	-	1	1	-	-	1	-	-	-	-	3	0.27
Trachea, bronchus and lung	C33-C34	-	-	-	1	1	1	4	9	7	10	3	3	2	1	1	-	43	3.88
Other thoracic organs	C37-C38	-	-	-	-	-	-	1	-	-	2	1	-	-	-	-	-	4	0.36
Bone	C40-C41	-	4	1	-	1	1	-	1	1	2	2	-	-	-	-	-	13	1.17
Other skin	C44	-	-	-	-	1	3	3	2	3	2	5	4	4	4	2	-	33	2.98
Connective and soft tissue	C47, C49	1	-	-	1	1	2	4	1	1	2	2	-	-	-	-	-	15	1.35
Breast	C50	-	1	1	5	18	24	25	30	26	15	14	9	6	3	-	-	177	15.97
Vulva	C51	-	-	-	-	-	-	-	1	-	-	-	-	-	-	-	-	1	0.09
Vagina	C52	-	-	1	1	-	1	-	-	-	-	1	-	-	-	-	-	4	0.36
Cervix uteri	C53	-	1	3	21	45	62	76	81	63	41	26	26	5	4	-	1	455	41.06
Corpus uteri	C54	-	-	1	-	1	1	2	1	4	2	2	-	-	-	-	-	14	1.26
Uterus unspecified	C55	-	-	-	-	1	2	-	3	2	2	2	-	-	-	-	-	12	1.08
Ovary	C56	-	2	2	2	4	-	4	3	2	2	1	-	1	-	-	1	24	2.17
Other female genital Organs	C57	-	-	-	-	-	-	-	2	2	-	-	-	-	-	-	-	4	0.36
Placenta	C58	-	-	-	-	-	1	-	-	-	-	-	-	-	-	-	-	1	0.09
Kidney	C64	-	-	1	2	1	1	1	-	1	1	2	-	-	-	-	-	10	0.90
Renal pelvis	C65	-	-	-	-	-	1	-	-	-	-	-	-	-	-	-	-	1	0.09
Bladder	C67	-	-	-	-	-	-	-	-	1	1	1	-	1	-	-	1	5	0.45
Other urinary organs	C68	-	-	-	-	-	-	-	-	-	1	-	-	-	-	-	-	1	0.09
Eye	C69	-	-	-	1	-	-	2	-	-	-	-	-	-	-	-	-	3	0.27
Brain, nervous system	C70-C72	-	-	1	1	3	1	-	-	2	1	-	1	-	-	-		10	0.90
Thyroid	C73	-	1	2	1	1	1	-	1	-	0	0	3	1	-	-	-	11	0.99
Other and unspecified	O&U	1	-	5	3	7	4	5	5	8	5	7	6	2	-	-	-	58	5.23
	Total	2	10	22	47	91	121	144	171	158	117	99	66	32	18	7	3	1,108	100

### Spatial distribution of cancer cases

**Fig. 2:**
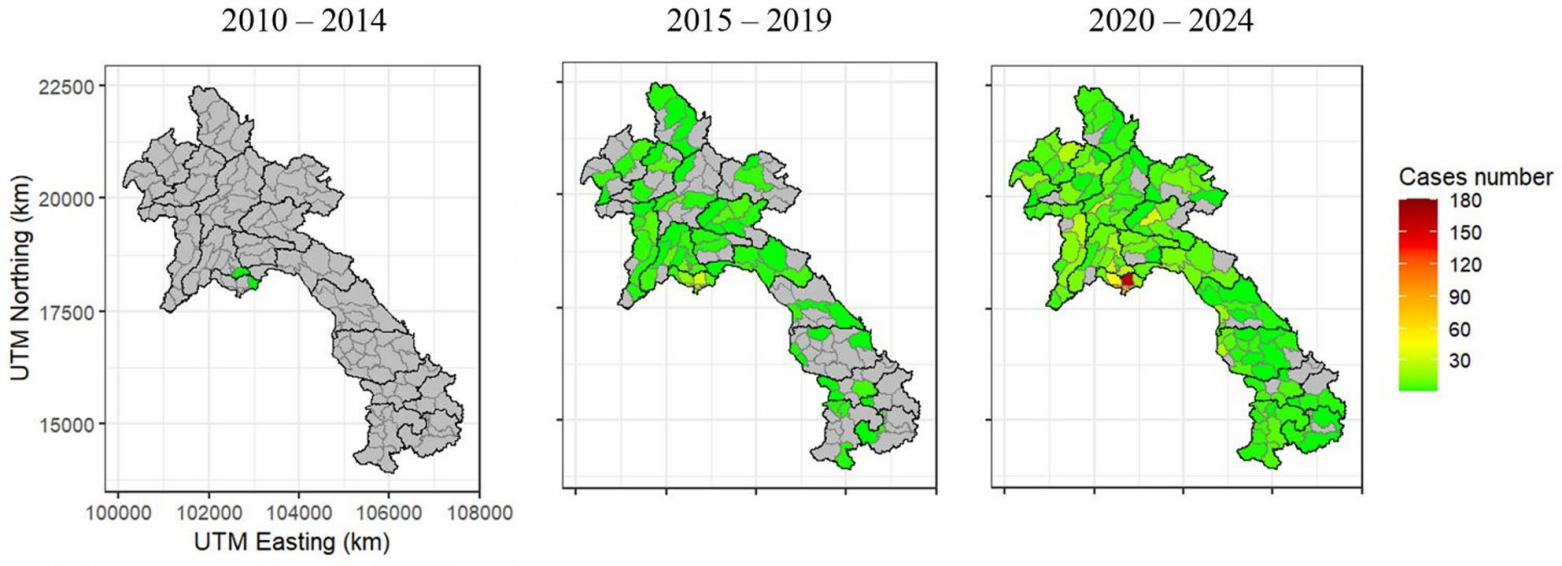
Spatial distribution by district of recorded cancer cases in Laos between 2010 and 2024. The Universal Transverse Mercator (UTM) coordinates are measured as northings and eastings in kilometers (km). The districts colored in gray had no recorded cases

Figure 2 shows the spatial distribution of cancer cases recorded at the National Cancer Center across provinces in Laos over three time periods (2010–2014, 2015–2019, and 2020–2024). The northern provinces, including Phongsaly, Oudomxai, and Louangnamtha, had no reported cases in earlier time periods but had improved case reporting by 2020–2024. Central provinces, including Vientiane Capital and surrounding areas, demonstrated the most consistent case reporting across all periods, likely reflecting better healthcare infrastructure and cancer-record systems in the capital region.

The southern provinces, such as Champasak and Attapeu, showed gradual improvement in case identification over time. The progression from predominantly gray (no recorded cases) in 2010–2014 to widespread green and yellow coloring in 2020–2024 across most provinces indicates significant strengthening of Laos’ national cancer surveillance system and healthcare capacity. This pattern indicates that cancer cases were previously undetected or unreported in many provinces rather than genuinely absent, highlighting the critical importance of developing diagnostic capabilities and cancer registries throughout the country (Fig. 2).

**Fig. 3:**
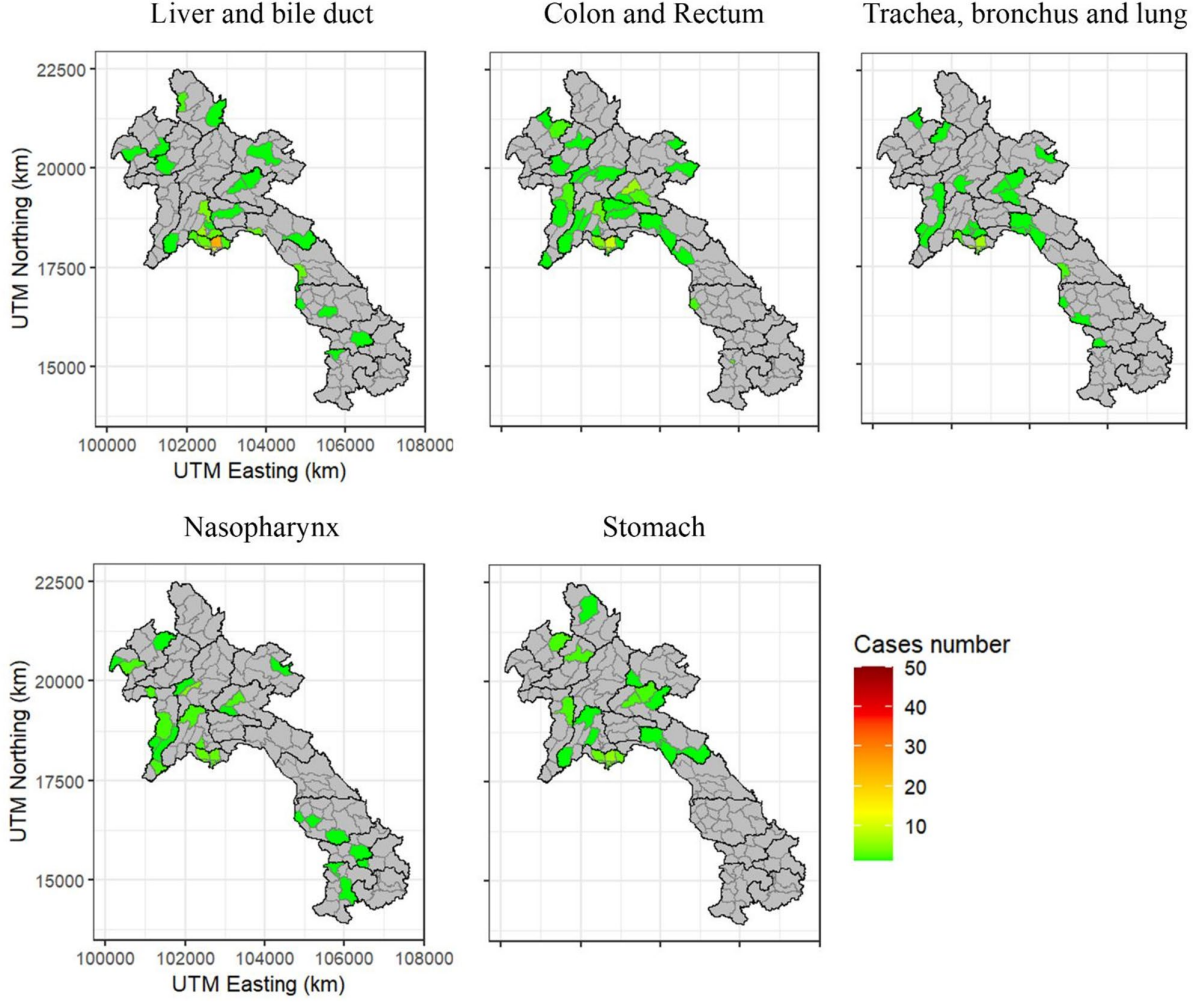
Most frequent cancers of males by district between 2010 and 2024

Figure 3 shows the distribution of the top five male cancers by district in Laos between 2010 and 2024, using a color scale from gray (no cases) to red (up to 50 cases). Liver and bile-duct cancer was recorded across multiple provinces, with the highest concentrations in northern and central regions, including Vientiane Capital. Colon and rectum cancers were more geographically restricted, occurring primarily in central provinces near the capital. Cancers of the trachea, stomach, bronchus, and lungs were recorded mainly in northern and central areas, while nasopharynx cancer was seen in certain northern provinces.

**Fig. 4:**
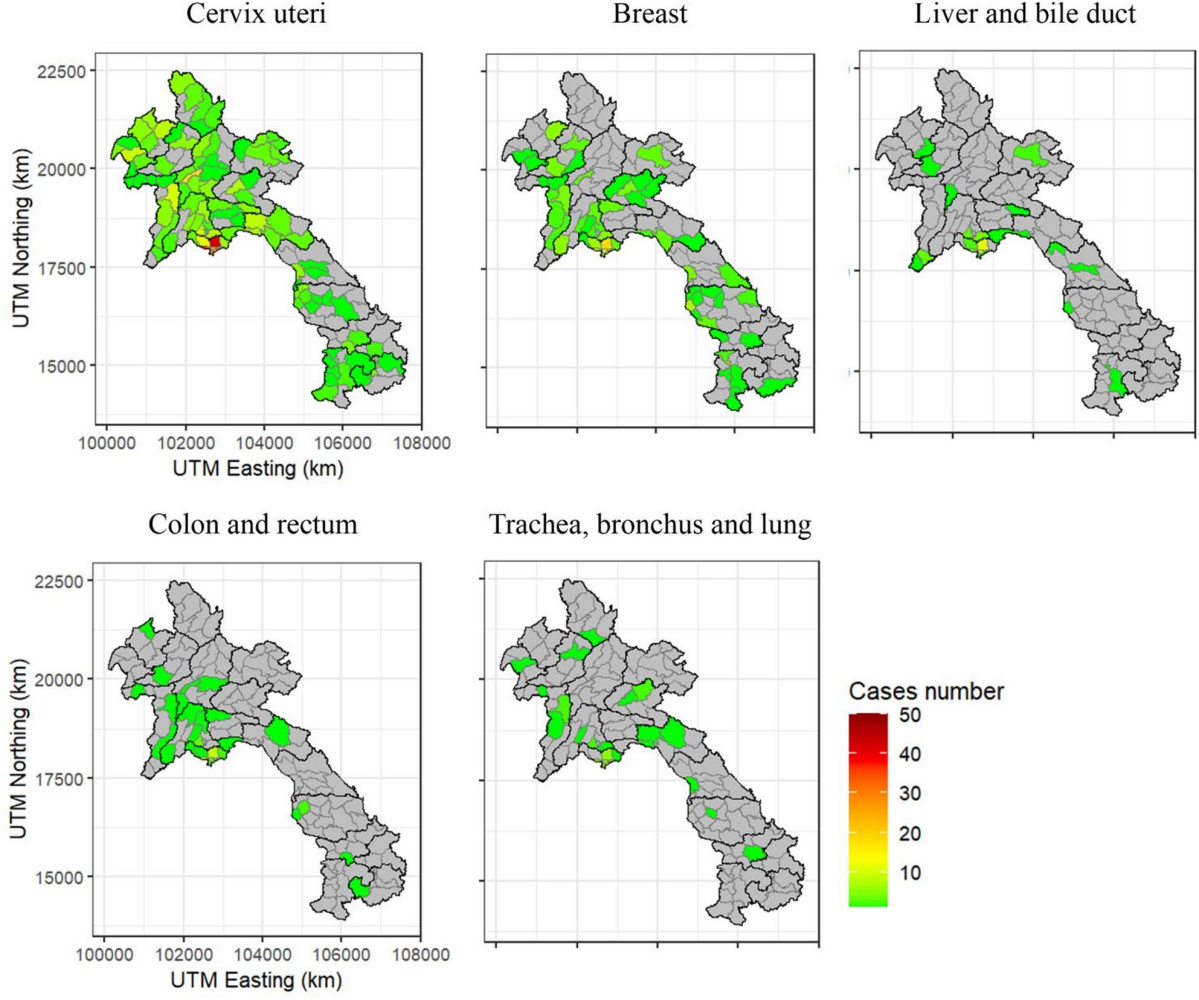
Most frequent cancers of females by district between 2010 and 2024

Figure 4 highlights that cervical cancer is a major public health problem throughout Laos. The yellow to orange coloring in many districts reflects its high prevalence and status as the most common cancer among women in the country. Breast cancer showed a more limited distribution, being primarily concentrated in central provinces around Vientiane province and some northern areas, with most districts showing green coloring indicating lower case numbers. Liver and bile-duct cancer appeared sporadically across various provinces, with relatively few reporting districts. Colorectal cancer had a scattered distribution, appearing mainly in central and some peripheral provinces. In contrast, cancers of the trachea, bronchus, and lung showed a more limited geographic spread, with cases concentrated in central regions and a few northern areas.

## Discussion

In this study, we followed IARC guidelines for data registration to ensure that our results are standardized and reliable [15, 16]. Reported cancer incidence in Lao PDR has doubled over the last two decades and is expected to increase further by 2040 [17]. This highlights that cancer needs to be regarded as a public health priority in the Lao PDR. Our 15-year analysis provides a critical foundational overview of the cancer landscape in Laos, highlighting a significant burden driven by preventable, infection-associated malignancies like liver and cervical cancer, alongside emerging lifestyle-related cancers such as those of the colon and breast. Our findings, while broadly consistent with regional reports [11], reveal unique local patterns and underscore a dual challenge: the high prevalence of these preventable cancers and the geographic disparities in diagnosis linked to healthcare access. This study also illuminates distinct gender-based patterns for cancer distribution across the Lao PDR.

The higher rate of histological confirmation in females (85.92%) compared to males (72.97%) reflects several underlying factors. First, the predominant cancer types differ substantially between sexes. Cervical cancer, which accounts for 41.1% of female cases, is highly accessible for tissue biopsy through routine gynecological examination and colposcopy. Similarly, breast cancer (15.97% of female cases) is amenable to core needle biopsy or excisional biopsy. In contrast, the leading male cancers—liver and bile-duct cancer (15.1%) and lung cancer (8.1%)—often present at advanced stages when patients are too frail for invasive diagnostic procedures. Also the National Cancer Center lacks facilities and expertise to diagnose these effectively and/or the tumor location makes biopsy technically challenging or contraindicated due to bleeding risk. Diagnosis in males showed higher reliance on endoscopy and radiology (19.24% vs. 7.31% in females), suggesting later-stage presentations where imaging findings are sufficiently characteristic to establish a clinical diagnosis without histological confirmation. The substantial proportion of cases lacking histological confirmation reflects constraints in diagnostic capacity in Lao PDR, including limited availability of pathology services outside the capital, insufficient biopsy equipment in peripheral hospitals, and financial barriers preventing patients from accessing specialized diagnostic procedures. These findings underscore the urgent need to strengthen pathology infrastructure and decentralize diagnostic services to improve cancer registry data quality nationwide. Predominance of infection-associated cancers is the major finding in the Lao PDR. This is a hallmark of cancer epidemiology in many parts of Southeast Asia [11, 18]. Among females, cervical cancer was overwhelmingly the most common malignancy, accounting for a staggering 41.1% of cases. This finding aligns with data from the GLOBOCAN 2020 [19], which identified cervical cancer as the most frequent cancer among women in Laos [6]. The primary cause of nearly all cervical cancers is persistent infection with high-risk strains of the human papillomavirus (HPV) [20]. The high incidence observed in our study reflects long-standing challenges in the country, including the limited availability of routine HPV vaccination for adolescents and a lack of widespread, systematic screening programs that can detect and treat precancerous lesions [12]. The geographic distribution of recorded cases, while reflecting patterns of healthcare access and referral to the National Cancer Center rather than true population-level incidence, suggests that cervical cancer represents a public health challenge extending beyond the capital region. However, the concentration of cases from Vientiane and surrounding provinces likely reflects both higher disease burden in areas with better diagnostic access and substantial under-ascertainment in remote provinces where women face barriers to screening and specialized care.

Among males, liver and bile-duct cancer was the leading malignancy (15.1%). This is consistent with patterns observed throughout the Greater Mekong Subregion, where liver cancer is a leading cause of cancer mortality [21]. The primary risk factors in Laos are highly prevalent and include chronic infection with the hepatitis B virus (HBV) and, to a lesser extent, the hepatitis C virus (HCV) [11]. Another significant contributor in the region is dietary exposure to aflatoxin, a mycotoxin produced by fungi that contaminates staple foods stored in humid conditions from the traditional agricultural practices, as in Vietnam [22]. Furthermore, the consumption of raw or undercooked freshwater fish can lead to infection with the liver fluke *Opisthorchis viverrini*, a known carcinogen that causes cholangiocarcinoma (bile-duct cancer), particularly in the southern provinces of Laos [23, 24]. The etiology and epidemiology of hepatocellular carcinoma (HCC) are changing across Asian countries, particularly in the Lao PDR, as well as worldwide [25]. The leading cause of liver-related death has shifted from chronic hepatitis B virus infection to metabolic dysfunction-associated steatotic liver disease (MASLD), which now affects an estimated 30% of adults. Alcohol-related liver disease is another fast-growing cause of liver cancer [9, 25, 26]. The relatively high incidence of nasopharynx cancer, particularly in males, is also characteristic of populations in Southern China and Southeast Asia. Risk factors are multifactorial and include genetic predisposition, consumption of salt-preserved foods, and infection with the Epstein-Barr virus (EBV) [27].

Emerging lifestyle-related cancers are also highlighted in this study. While infection-related cancers dominate, our data also show a notable incidence of other malignancies that may signify an emerging epidemiological transition. Colorectal cancer was the second most common cancer in males (11.45%), and breast cancer was the second leading cancer in females (15.97%). This trend may reflect a gradual shift where dietary and lifestyle factors such as increased consumption of processed foods, higher meat intake, and decreased physical activity are becoming more prevalent, a pattern seen in other developing economies in the region [28]. This trend of increasing colorectal cancer in the Lao PDR reflects trends in other Asian countries [29].

Our findings align closely with cancer patterns observed in neighboring Greater Mekong Subregion countries, particularly Thailand, Vietnam, and Cambodia, which share similar risk factor profiles and epidemiological transitions [30, 31]. In Thailand, liver cancer remains among the top three cancers in males, with particularly high incidence in northeastern provinces where *Opisthorchis viverrini* infection is endemic—a pattern mirroring our observations in Lao PDR, given the shared cultural practices of consuming raw fish dishes [32, 33]. Similarly, cervical cancer continues to be a leading female malignancy across the region despite the availability of HPV vaccination and screening programs in some countries, reflecting persistent challenges in achieving high coverage, particularly in rural populations [34]. Vietnam has documented a similar dual burden, with infection-related cancers (liver, cervical, gastric) coexisting with rising rates of lifestyle-related malignancies (colorectal, breast) as the country undergoes rapid economic development [35]. Cambodia, which shares Lao PDR’s low- to middle-income status and limited cancer surveillance infrastructure, reports comparable difficulties in accurately estimating cancer burden due to reliance on hospital-based data and modeled estimates [2, 36]. The success of population-based registries in neighboring Thailand, particularly in Chiang Mai and Khon Kaen, provides clear evidence that strengthening surveillance is attainable even in resource-limited environments. These systems yield the high-quality data required to drive effective cancer control strategies and public health policy [37, 38]. The regional consistency of these patterns reinforces that the cancers we observe are not unique to Lao PDR but rather reflect shared environmental, infectious, behavioral, and structural determinants that require coordinated regional prevention strategies alongside national investments in diagnosis and treatment capacity [39, 40].

Healthcare system development, elimination of disparities, and filling of data gaps are required in the Lao PDR. Our spatial and temporal analysis provides a compelling narrative of a strengthening national health system. The marked increase in case reporting from northern and southern provinces over the 15 years likely reflects improved diagnostic capabilities and the expansion of the cancer record system rather than a true explosion in cancer incidence. Critically, the geographic distribution of recorded cases reflects healthcare access and referral patterns rather than cancer incidence. The concentration of cases in Vientiane Capital reflects the centralization of diagnostic services at the National Cancer Center. Rural provinces remain severely under-represented due to geographic distance, costs, and limited diagnostic capacity. This referral bias systematically underestimates the cancer burden outside the capital.

However, the geographic distribution of records also highlights stark inequalities. The concentration of diverse cancer types in and around Vientiane Capital suggests a highly centralized healthcare system where specialized diagnostic services are not yet nationally available. This implies that the true cancer burden in rural areas is significantly underestimated. The diagnostic methods used also reflect this. The heavy reliance on histology for females (85.92%) is logical, as the leading cancers (cervical and breast) are highly accessible for biopsy. In contrast, the high use of “history and physical examination” for males (19.21%) may suggest that males present with more advanced disease or that there is greater difficulty in accessing definitive diagnostic procedures [25].

The most striking finding of this study is not what it reveals, but what it fails to capture. Our dataset contains only 1,738 cancer cases recorded over 15 years (2010–2024), averaging approximately 116 cases per year, with patient age grouped into 16 categories using 5-year intervals [41]. This stands in stark contrast to GLOBOCAN 2020 estimates projecting approximately 9,133 new cancer cases annually in Lao PDR [6]. This means our hospital-based registry captures only about 1.3% of the estimated national cancer burden per year. This enormous discrepancy represents a fundamental limitation that constrains all interpretations of our findings. The reasons for this severe under-ascertainment are multifactorial: (1) The National Cancer Center is a single tertiary facility in the capital, and many patients never reach specialized cancer care due to geographic, financial, and cultural barriers; (2) Advanced diagnostic capacity (histopathology, imaging) remains highly centralized, leaving most peripheral health facilities unable to definitively diagnose cancer; (3) Many patients, particularly in rural areas, may present to traditional healers or remain undiagnosed until death; (4) The absence of a systematic population-based cancer registry means cases diagnosed elsewhere, or not diagnosed at all, remain invisible to the health system. Therefore, our data should be understood as representing a small, highly selected subset of Lao PDR’s true cancer burden; specifically, those patients with sufficient resources, awareness, and access to reach the National Cancer Center. Any spatial, temporal, or epidemiological patterns we describe must be interpreted with this profound selection bias in mind. The urgent implication is that Lao PDR faces a largely invisible cancer epidemic, with the vast majority of cases occurring, progressing, and resulting in death without formal diagnosis or treatment. Establishing a national population-based cancer registry is not merely desirable but essential to understanding and addressing the true scale of this public health crisis.

The low reported incidence of the top five cancers in southern Lao PDR in Figs. 3 and 4 is likely due to incomplete case detection and underdiagnosis caused by limitations in the health system and cancer surveillance, rather than a truly low cancer risk in this region [42]. The main cause of this geographical discrepancy is the centralized healthcare infrastructure, which makes it impossible to formally identify cases in rural southern provinces due to the concentration of pathology services and diagnostic equipment in Vientiane Capital [43]. Additionally, the Lao PDR’s hospital-based registries do not include the data of southern province residents who often avoid local hospitals in favor of seeking specialized oncology care across the border in Thailand (e.g., Khon Kaen or Ubon Ratchathani) [44]. These factors, compounded by lower health literacy and the high “indirect costs” of travel, suggest that southern “cold spots” on spatial maps represent gaps in diagnostic capacity and surveillance rather than a lower demographic or environmental risk [45, 46].

This study is subject to the limitations inherent to its reliance on hospital-based record data, which results in a lower number of patients compared to the cancer registry reported in GLOBOCAN. As the data are not population-based, they cannot be used to calculate true incidence rates and are susceptible to selection bias, capturing only patients who access the National Cancer Center. The lack of histological diagnoses for liver and lung cancer highlights the need to definitively distinguish between primary tumors and metastases, and points to challenges in diagnostic capacity. Therefore, the true national cancer burden is likely much higher than what is reported here. Despite these limitations, this study transforms a raw dataset into actionable intelligence, providing an invaluable baseline for understanding cancer epidemiology in Lao PDR. Furthermore, the retrospective nature of the study did not allow patients to be questioned about their lifestyle habits (alcohol and tobacco consumption), family history of cancer etc.

## Conclusion

In conclusion, this analysis of hospital-based cancer records from the National Cancer Center reveals patterns dominated by preventable, infection-associated malignancies, particularly cervical cancer in females and liver cancer in males. However, these findings must be interpreted with careful recognition that they reflect healthcare access patterns and referral pathways to a single tertiary center rather than true population-level cancer incidence across Lao PDR. The most common cancers are liver cancer in males and cervical and breast cancer in females. The spatial distribution of recorded cases is heavily skewed toward the national capital, indicating major disparities in healthcare access and surveillance capacity across the country. Strengthening the national cancer-reporting system, expanding decentralized diagnostic services, and implementing targeted prevention programs for known infectious agents causing cervical and liver cancer are urgent priorities for improving cancer control in the country.

## Data Availability

No datasets were generated or analysed during the current study.

## References

[1] Bray F, Ferlay J, Soerjomataram I, Siegel RL, Torre LA, Jemal A. Global cancer statistics 2018: GLOBOCAN estimates of incidence and mortality worldwide for 36 cancers in 185 countries. CA Cancer J Clin. 2018;68(6):394–424. 30207593 10.3322/caac.21492

[2] Ferlay J, Colombet M, Soerjomataram I, Parkin DM, Pineros M, Znaor A, Bray F. Cancer statistics for the year 2020: An overview. Int J Cancer. 2021;149(4):1–12. 10.1002/ijc.33588 10.1002/ijc.33588 33818764

[3] World Health Organization. Noncommunicable diseases country profiles 2018. [Internet].Geneva: World Health Organization; 2018 [cited 2026 Jan 9]. Available from: https://www.who.int/publications/i/item/ncd-country-profiles-2018.

[4] Ferlay J, Colombet M, Soerjomataram I, Mathers C, Parkin DM, Pineros M, Znaor A, Bray F. Estimating the global cancer incidence and mortality in 2018: GLOBOCAN sources and methods. Int J Cancer. 2019;144(8):1941–53. 30350310 10.1002/ijc.31937

[5] Study GBoD. Global burden of disease study 2019 results. In.: Institute for Health Metrics and Evaluation; 2020. http://ghdx.healthdata.org/gbd-results-tool.

[6] Sung H, Ferlay J, Siegel RL, Laversanne M, Soerjomataram I, Jemal A, Bray F. Global Cancer Statistics 2020: GLOBOCAN Estimates of Incidence and Mortality Worldwide for 36 Cancers in 185 Countries. CA Cancer J Clin. 2021;71(3):209–49. 33538338 10.3322/caac.21660

[7] McGlynn KA, Petrick JL, El-Serag HB. Epidemiology of Hepatocellular Carcinoma. Hepatology. 2021;73(1Suppl 1):4–13. 32319693 10.1002/hep.31288 PMC7577946

[8] Caines A, Selim R, Salgia R. The Changing Global Epidemiology of Hepatocellular Carcinoma. Clin Liver Dis. 2020;24(4):535–47. 33012444 10.1016/j.cld.2020.06.001

[9] Sarin SK, Kumar M, Eslam M, George J, Al Mahtab M, Akbar SMF, Jia J, Tian Q, Aggarwal R, Muljono DH, et al. Liver diseases in the Asia-Pacific region: a Lancet Gastroenterology & Hepatology Commission. Lancet Gastroenterol Hepatol. 2020;5(2):167–228. 31852635 10.1016/S2468-1253(19)30342-5 PMC7164809

[10] Singal AG, Kanwal F, Llovet JM. Global trends in hepatocellular carcinoma epidemiology: implications for screening, prevention and therapy. Nat Rev Clin Oncol. 2023;20(12):864–84. 37884736 10.1038/s41571-023-00825-3

[11] Sitbounlang P, Marchio A, Deharo E, Paboriboune P, Pineau P. The Threat of Multiple Liver Carcinogens in the Population of Laos: A Review. Livers vol. 2021;1:49–59.

[12] Phaiphichit J, Paboriboune P, Kunnavong S, Chanthavilay P. Factors associated with cervical cancer screening among women aged 25–60 years in Lao People’s Democratic Republic. PLoS ONE. 2022;17(4):e0266592. 35390098 10.1371/journal.pone.0266592 PMC8989294

[13] Parkin DM. The evolution of the population-based cancer registry. Nat Rev Cancer. 2006;6(8):603–12. 16862191 10.1038/nrc1948

[14] Bray F, Parkin DM. Evaluation of data quality in the cancer registry: principles and methods. Part I: comparability, validity and timeliness. Eur J Cancer. 2009;45(5):747–55. 19117750 10.1016/j.ejca.2008.11.032

[15] Bray F, Laversanne M, Sung H, Ferlay J, Siegel RL, Soerjomataram I, Jemal A. Global cancer statistics 2022: GLOBOCAN estimates of incidence and mortality worldwide for 36 cancers in 185 countries. CA Cancer J Clin. 2024;74(3):229–63. 38572751 10.3322/caac.21834

[16] Filho AM, Laversanne M, Ferlay J, Colombet M, Pineros M, Znaor A, Parkin DM, Soerjomataram I, Bray F. The GLOBOCAN 2022 cancer estimates: Data sources, methods, and a snapshot of the cancer burden worldwide. Int J Cancer. 2025;156(7):1336–46. 39688499 10.1002/ijc.35278

[17] Mousavi SE, Ilaghi M, Aflatoonian S, Nejadghaderi SA. Epidemiology, socioeconomic correlates, and trend projections of multiple myeloma in Asia over 2020–2040. Heliyon. 2025;11(9):e43325.

[18] Feliciano EJG, Ho FDV, Yee K, Paguio JA, Eala MAB, Robredo JPG, Ng K, Lim J, Pyone KT, Peralta CA, et al. Cancer disparities in Southeast Asia: intersectionality and a call to action. Lancet Reg Health West Pac. 2023;41:100971. 38053740 10.1016/j.lanwpc.2023.100971 PMC10694578

[19] Wu J, Jin Q, Zhang Y, Ji Y, Li J, Liu X, Duan H, Feng Z, Liu Y, Zhang Y, et al. Global burden of cervical cancer: current estimates, temporal trend and future projections based on the GLOBOCAN 2022. J Natl Cancer Cent. 2025;5(3):322–9. 40693230 10.1016/j.jncc.2024.11.006 PMC12276544

[20] Phongsavan K, Gustavsson I, Marions L, Phengsavanh A, Wahlstrom R, Gyllensten U. Detection of Human Papillomavirus Among Women in Laos: Feasibility of Using Filter Paper Card and Prevalence of High-Risk Types. Int J Gynecol Cancer. 2012;22(8):1398–406. 22932265 10.1097/IGC.0b013e3182664b6b

[21] McGuire S. World Cancer Report 2014. Geneva, Switzerland: World Health Organization, International Agency for Research on Cancer, WHO Press, 2015. Adv Nutr. 2016;7(2):418–419. 10.3945/an.116.012211 PMC4785485 26980827

[22] Phan LTK, Tran TM, De Boevre M, Jacxsens L, Eeckhout M, De Saeger S. Impact of Season, Region, and Traditional Agricultural Practices on Aflatoxins and Fumonisins Contamination in the Rice Chain in the Mekong Delta, Vietnam. Toxins (Basel). 2021;13(9):667. 10.3390/toxins13090667 PMC8473189 34564671

[23] Sithithaworn P, Andrews RH, Nguyen VD, Wongsaroj T, Sinuon M, Odermatt P, Nawa Y, Liang S, Brindley PJ, Sripa B. The current status of opisthorchiasis and clonorchiasis in the Mekong Basin. Parasitol Int. 2012;61(1):10–6. 21893213 10.1016/j.parint.2011.08.014 PMC3836690

[24] Sripa B, Suwannatrai AT, Sayasone S, Do DT, Khieu V, Yang Y. Current status of human liver fluke infections in the Greater Mekong Subregion. Acta Trop. 2021;224:106133. 34509453 10.1016/j.actatropica.2021.106133

[25] Kim DY. Changing etiology and epidemiology of hepatocellular carcinoma: Asia and worldwide. J Liver Cancer. 2024;24(1):62–70. 38523466 10.17998/jlc.2024.03.13 PMC10990659

[26] Mak LY, Liu K, Chirapongsathorn S, Yew KC, Tamaki N, Rajaram RB, Panlilio MT, Lui R, Lee HW, Lai JC, et al. Liver diseases and hepatocellular carcinoma in the Asia-Pacific region: burden, trends, challenges and future directions. Nat Rev Gastroenterol Hepatol. 2024;21(12):834–51. 39147893 10.1038/s41575-024-00967-4

[27] Jia WH, Qin HD. Non-viral environmental risk factors for nasopharyngeal carcinoma: a systematic review. Semin Cancer Biol. 2012;22(2):117–26. 22311401 10.1016/j.semcancer.2012.01.009

[28] Center MM, Jemal A, Ward E. International trends in colorectal cancer incidence rates. Cancer Epidemiol Biomarkers Prev. 2009;18(6):1688–94. 19505900 10.1158/1055-9965.EPI-09-0090

[29] Pardamean CI, Sudigyo D, Budiarto A, Mahesworo B, Hidayat AA, Baurley JW. Pardamean B: Changing Colorectal Cancer Trends in Asians: Epidemiology and Risk Factors. Oncol Rev. 2023;17:10576. 37284188 10.3389/or.2023.10576 PMC10241074

[30] Moore MA, Attasara P, Khuhaprema T, Le TN, Nguyen TH, Raingsey PP, Sriamporn S, Sriplung H, Srivanatanakul P, Bui DT, et al. Cancer epidemiology in mainland South-East Asia - past, present and future. Asian Pac J Cancer Prev. 2010;11(Suppl 2):67–80. 20553069

[31] Pakharukova MY, Mordvinov VA. The liver fluke *Opisthorchis felineus*: biology, epidemiology and carcinogenic potential. Trans R Soc Trop Med Hyg. 2016;110(1):28–36. 26740360 10.1093/trstmh/trv085

[32] Sriamporn S, Pisani P, Pipitgool V, Suwanrungruang K, Kamsa-ard S, Parkin DM. Prevalence of *Opisthorchis viverrini* infection and incidence of cholangiocarcinoma in Khon Kaen, Northeast Thailand. Trop Med Int Health. 2004;9(5):588–94. 15117303 10.1111/j.1365-3156.2004.01234.x

[33] Vatanasapt V, Uttaravichien T, Mairiang EO, Pairojkul C, Chartbanchachai W, Haswell-Elkins M. Cholangiocarcinoma in north-east Thailand. Lancet. 1990;335(8681):116–7. 1967406 10.1016/0140-6736(90)90591-r

[34] Chansaenroj J, Lurchachaiwong W, Termrungruanglert W, Tresukosol D, Niruthisard S, Trivijitsilp P, Sampatanukul P, Poovorawan Y. Prevalence and genotypes of human papillomavirus among Thai women. Asian Pac J Cancer Prev. 2010;11(1):117–22. 20593940

[35] Pham T, Bui L, Kim G, Hoang D, Tran T, Hoang M. Cancers in Vietnam-Burden and Control Efforts: A Narrative Scoping Review. Cancer Control. 2019;26(1):1073274819863802. 31319695 10.1177/1073274819863802 PMC6643189

[36] Dee EC, Laversanne M, Bhoo-Pathy N, Ho FDV, Feliciano EJG, Eala MAB, Ting FIL, Ginsburg O, Moraes FY, Gyawali B, et al. Cancer incidence and mortality estimates in 2022 in southeast Asia: a comparative analysis. Lancet Oncol. 2025;26(4):516–28. 40024257 10.1016/S1470-2045(25)00017-8

[37] Insamran W, Sangrajrang S. National Cancer Control Program of Thailand. Asian Pac J Cancer Prev. 2020;21(3):577–82. 32212781 10.31557/APJCP.2020.21.3.577 PMC7437310

[38] Sriplung H, Wiangnon S, Sontipong S, Sumitsawan Y, Martin N. Cancer incidence trends in Thailand, 1989–2000. Asian Pac J Cancer Prev. 2006;7(2):239–44. 16839216

[39] Suwanrungruang K, Sriamporn S, Wiangnon S, Rangsrikajee D, Sookprasert A, Thipsuntornsak N, Satitvipawee P, Poomphakwaen K, Tokudome S. Lifestyle-related risk factors for stomach cancer in northeast Thailand. Asian Pac J Cancer Prev. 2008;9(1):71–5. 18439078

[40] Torre LA, Siegel RL, Ward EM, Jemal A. Global Cancer Incidence and Mortality Rates and Trends–An Update. Cancer Epidemiol Biomarkers Prev. 2016;25(1):16–27. 26667886 10.1158/1055-9965.EPI-15-0578

[41] Sung H, Siegel RL, Laversanne M, Jiang C, Morgan E, Zahwe M, Cao Y, Bray F, Jemal A. Colorectal cancer incidence trends in younger versus older adults: an analysis of population-based cancer registry data. Lancet Oncol. 2025;26(1):51–63. 39674189 10.1016/S1470-2045(24)00600-4 PMC11695264

[42] Agbedinu K, Antwi S, Aduse-Poku L, Akakpo PK, Larrious-Lartey H, Ofori Aboah V, Mensah S, Nyarko V, Amponsah-Manu F, Nsaful J, et al. A Scoping Review on Barriers to Cancer Diagnosis and Care in Low- and Middle-Income Countries. Cancer Epidemiol Biomarkers Prev. 2025;34(7):1066–73. 40304503 10.1158/1055-9965.EPI-25-0120

[43] Retrospective Appraisal of Cancer Patients from Vientiane Capital City. Lao People’s Democratic Republic (PDR), Seeking Treatment in Thailand. Asian Pac J Cancer Prev. 2013;14(9):5435–40. 24175839 10.7314/apjcp.2013.14.9.5435

[44] Bochaton A. Cross-border mobility and social networks: Laotians seeking medical treatment along the Thai border. Soc Sci Med. 2015;124:364–73. 25454637 10.1016/j.socscimed.2014.10.022

[45] Bodhisane S, Pongpanich S. The accessibility and probability of encountering catastrophic health expenditure by Lao patients in Thai hospitals. J Public Health. 2021;44(2):457–70. 10.1093/pubmed/fdab043 33895842

[46] Alberto NRI, Alberto IRI, Puyat CVM, Antonio MAR, Ho FDV, Dee EC, Mahal BA, Eala MAB. Disparities in access to cancer diagnostics in ASEAN member countries. Lancet Reg Health West Pac. 2023;32:100667. 36785859 10.1016/j.lanwpc.2022.100667 PMC9918780

